# Two-Dimensional DOA Estimation for Three-Parallel Nested Subarrays via Sparse Representation

**DOI:** 10.3390/s18061861

**Published:** 2018-06-07

**Authors:** Weijian Si, Zhanli Peng, Changbo Hou, Fuhong Zeng

**Affiliations:** College of Information and Communication Engineering, Harbin Engineering University, No. 145 Nantong Street, Harbin 150001, China; swj0418@263.net (W.S.); zhanlipeng9@gmail.com (Z.P.); fuhongzeng@gmail.com (F.Z.)

**Keywords:** two-dimensional DOA estimation, three-parallel nested subarrays, sparse representation, cross-correlation matrix, degrees of freedom

## Abstract

Nested arrays are considered attractive due to their hole-free performance, and have the ability to resolve $O\left(N^{2}\right)$ sources with O(N) physical sensors. Inspired by nested arrays, two kinds of three-parallel nested subarrays (TPNAs), which are composed of three parallel sparse linear subarrays with different inter-element spacings, are proposed for two-dimensional (2-D) direction-of-arrival (DOA) estimation in this paper. We construct two cross-correlation matrices and combine them as one augmented matrix in the first step. Then, by vectorizing the augmented matrix, a hole-free difference coarray with larger degrees of freedom (DOFs) is achieved. Meanwhile, sparse representation and the total least squares technique are presented to transform the problem of 2-D DOA searching into 1-D searching. Accordingly, we can obtain the paired 2-D angles automatically and improve the 2-D DOA estimation performance. In addition, we derive closed form expressions of sensor positions, as well as number of sensors for different subarrays of two kinds of TPNA to maximize the DOFs. In the end, the simulation results verify the superiority of the proposed TPNAs and 2-D DOA estimation method.

## 1. Introduction

Direction of arrival (DOA) estimation of multiple far-field narrowband sources is a vital problem in the field of array signal processing and is widely used in radar, sonar, radio astronomy, wireless communications [1], etc. Over the past few decades, many methods, such as multiple signal classification (MUSIC) [2] and estimation of signal parameters via rotational invariance technique (ESPRIT) [3], have been presented to estimate the DOAs. Among these methods, however, uniform arrays whose inter-element spacing is less than or equal to the half-wavelength are required, such as the uniform linear array (ULA) [4], uniform rectangular array (URA) [5] and uniform circular array (UCA) [6]. Accordingly, the detection capability of the existing methods is limited by the number of physical sensors. To obtain desirable estimation accuracy, massive sensors are needed. This will lead to high computational complexity and great hardware cost and is obviously inapplicable in practical situations.

To address these problems, various sparse arrays, such as minimum redundancy arrays (MRAs) [7], nested arrays (NAs) [8] and coprime arrays (CPAs) [9], have been proposed. In [7], by constructing an augmented covariance matrix, the degrees of freedom (DOFs) of MRAs are improved. Nevertheless, the lack of closed from expression for MRAs causes the optimal design of sensors position is not easy to obtain. In [8], the nested arrays, which comprise two uniform linear subarrays with different inter-element spacing, can provide $O\left(N^{2}\right)$ DOFs by using O(N) physical sensors. Furthermore, NAs are easy to construct due to the existence of the closed form expressions for the sensors positions. However, the mutual coupling [10,11] between sensors in dense subarrays for NAs can result in the degradation of estimation performance. CPAs were proposed in [9] to improve the estimation performance, which can achieve O(MN) DOFs with O(M+N) physical sensors, where M and N are coprime integers. One notes that, due to the existence of holes in different coarrays for CPAs, the number of continuous DOFs is smaller than that of NAs. Among these sparse arrays, the DOFs are obtained by vectorizing the auto-correlation matrix of received signal data. Meanwhile, the spatial smoothing technique [12,13,14] is applied to recover the full rank of the auto-covariance matrix for the corresponding single snapshot received signal data in the coarray domain. Then, subspace-based methods are used to perform DOA estimation. However, only half of the DOFs can be applied effectively. To overcome these aforementioned challenges, many effective sparse array geometries have been further developed, including super nested arrays [15,16], augmented nested arrays [17], generalized coprime arrays [18], and so on. To enhance the utilization of DOFs, methods such as matrix completion [19,20] and sparse representation [21,22] have been proposed. Unfortunately, all of the above-mentioned array configurations and methods are focused on one-dimensional (1-D) DOA estimation.

In recent years, the problem of two-dimensional (2-D) DOA estimation for sparse arrays has drawn extensive attention due to the advantage of being able to simultaneously estimate more azimuths and elevations than the number of sensors. Some well-known 2-D sparse array geometries such as 2-D nested arrays [23,24] and hourglass arrays [25] have been proposed. They possess merits similar to 1-D nested arrays, i.e., $O\left(N^{2}\right)$ DOFs can be acquired with O(N) physical sensors. With the use of an auto-correlation matrix for received signal data, the 2-D DOA can be accurately estimated. However, only the spatial smoothing MUSIC algorithm and 2-D unitary ESPRIT algorithm have been introduced in the above-mentioned 2-D sparse array geometries. Their respective available DOFs cannot be fully used for the 2-D DOA estimation problem. In addition, several sparse parallel arrays with attractive performance have also been proposed that could be used to tackle the 2-D DOA estimation problem as two separate 1-D DOA estimation ones by using the parallel property of these sparse arrays. In [26], a parallel coprime array with predefined shift distance among two subarrays was proposed. By using M+N−1 sensors to construct this configuration, MN+M−1 continuous DOFs are obtained. However, only the positive part of the difference coarray was considered in [26], which does not fully exploit the advantages of coprime property. So as to achieve higher DOFs, some modified versions, including parallel coprime subarrays (PCPA) [27], symmetric coprime parallel arrays (S-CPPA) [28] and three-parallel co-prime arrays (TPCPA) [29], have been successively proposed. They can dramatically increase the continuous DOFs with systematical design and analysis. With these, however, the holes still exist under the coarray equivalence, limiting the increase of continuous DOFs, and the coarray cannot be fully used in the 2-D DOA estimation problem.

In this paper, we propose two kinds of novel three-parallel nested subarrays (TPNAs), which possess the advantages of 1-D nested arrays, such as a hole-free coarray property and larger DOFs compared to a parallel coprime array and its modifications. They are comprised of three parallel subarrays, which respectively contain $N_{1}$, $N_{2}$ and $N_{2}$ sensors with different inter-element spacings. Although the effects of mutual coupling are not considered in this paper, we separate the dense subarray that with $N_{1}$ sensors into two parts, each of which possesses double the inter-element unit spacing to reduce the mutual coupling effect. By constructing two cross-correlation matrices from the subarray measurement data, the effect of noise can be eliminated to some extent. Then, we combine these two cross-correlation matrices into one enhanced matrix. With the use of vectorization, one augmented virtual array with hole-free property is achieved, and the 2-D DOA estimation problem is transformed into two separate 1-D DOA estimation problems naturally. Finally, we propose to use the sparse representation framework [26,27,28,30] and total least squares technique [28,31] to estimate the paired 2-D angle parameters accurately and automatically. In this way, up to $O\left(N^{2}\right)$ sources can be resolved effectively with O(N) sensors, where $N=N_{1}+2N_{2}$. Numerical simulation results verify the superior of the proposed configurations and method.

The main contributions of this paper can be summarized as follows:

- We propose two kinds of TPNAs so as to estimate more sources than the number of sensors in the 2-D DOA estimation problem. The closed form expressions of sensors positions are presented, and the number of sensors for different subarrays for TPNAs is investigated to achieve the maximization of continuous DOFs.
- The two proposed kinds of TPNAs can achieve $O\left(N^{2}\right)$ continuous DOFs with O(N) sensors by using the vectorization operation of cross-correlation matrices, which are larger than a parallel co-prime array or its modifications.
- By optimizing the sensor distributions in two kinds of TPNAs, the effects of mutual coupling are moderately alleviated.
- By using the sparse representation framework and total least squares technique, the 2-D DOA estimation problem is decomposed into two 1-D DOA estimation problems, and the paired DOAs can be obtained automatically. In addition, the continuous DOFs can be fully used via the sparse representation framework.

The rest of this paper is organized as follows. The system model used in this paper is briefly introduced in Section 2. In Section 3, we propose two kinds of TPNA configurations and demonstrate the achievable DOFs as well as the corresponding optimal sensor positions. Then, the sparse representation framework is presented to estimate the normalized azimuths, and the total least squares technique is presented to estimate the corresponding normalized elevations automatically. The results of several numerical simulations are shown in Section 4, while Section 5 gives the conclusion for this paper.

Notation: In this paper, scalars, vectors, matrices and sets are respectively denoted by italic lowercase letters (a), italic lowercase bold letters (a), italic capital bold letters (A) and capital letters in blackboard boldface (A). In particular, $I_{K}$ denotes the K×K identity matrix. ${\mathbb{Z}}^{+}$ is used to denote the set of positive integers. ${\left(.\right)}^{*}$, ${\left(.\right)}^{T}$ and ${\left(.\right)}^{H}$ denote the conjugation, transpose and conjugate transpose, respectively. ${\left(.\right)}^{-1}$ denotes the inverse. [.] round a number to the nearest integer. E[.] represents the expectation operator, vec(.) is used to represent the vectorization and the symbol ⊗ represents the Kronecker product. Let diag[a] stands for a diagonal matrix that uses the elements of a as its diagonal elements. The $l_{1}$-norm and $l_{2}$-norm are respectively denoted by ${\left\|.\right\|}_{1}$ and ${\left\|.\right\|}_{2}$.

## 2. System Model

**Figure 1:**
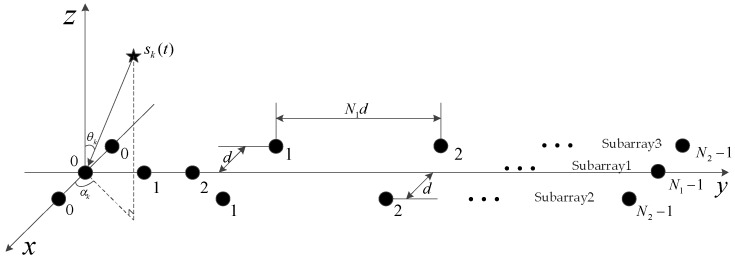
Illustration of the array geometry.

As illustrated in Figure 1, consider one of the three-parallel nested subarray configurations consisting of three parallel sparse linear subarrays lying on the *x*-*y* plane. The subarray1 has $N_{1}$ sensors lying on *y*-axis and the sensors’ positions relative to the *y*-axis can be expressed as $Y_{1}=\left\{y_{m}d|y_{m}\in \mathbb{Z},m=0,1,\cdots ,N_{1}-1\right\}$, whereas subarray2 and subarray3 along the *y*-axis have $N_{2}$ sensors with an inter-element spacing of $N_{1}d$, respectively. Then, the sensors’ positions of subarray2 and subaray3 along the *y*-axis can be expressed as $Y_{2}=Y_{3}=\left\{y_{n}N_{1}d|y_{n}\in \mathbb{Z},n=0,1,\cdots ,N_{2}-1\right\}$. Here, we assume $d=\frac{\lambda }{2}$ is the unit inter-element spacing, λ is the wavelength of the incident signal, the distance between subarray1 and subarray2 and that between subarray1 and subarray3 is set equal to d. Therefore, the total number of sensors in TPNAs is $N=N_{1}+2N_{2}$.

Suppose K far-field narrowband uncorrelated sources impinge on this array with unknown directions of arrival $\left(\alpha_{k},\theta_{k}\right),k=1,\cdots ,K$, where $\alpha_{k}\in \left[-\pi ,\pi \right]$ and $\theta_{k}\in \left[0,\frac{\pi }{2}\right]$ denote the azimuth angle and elevation angle of the *k*-th signal, respectively. To simplify the system model, we ignore the effects of mutual coupling in Figure 1. Then, the array measurement vectors of the three subarrays at the *t*-th snapshot can be expressed as

(1) $$x_{1}\left(t\right)=\overset{K}{\underset{k=1}{\sum }}a_{1}\left(\alpha_{k},\theta_{k}\right)s_{k}\left(t\right)+n_{1}\left(t\right)=A_{1}\left(\alpha ,\theta \right)s\left(t\right)+n_{1}\left(t\right),$$

(2) $$x_{2}\left(t\right)=\overset{K}{\underset{k=1}{\sum }}a_{2}\left(\alpha_{k},\theta_{k}\right)s_{k}\left(t\right)+n_{2}\left(t\right)=A_{2}\left(\alpha ,\theta \right)s\left(t\right)+n_{2}\left(t\right),$$

(3) $$x_{3}\left(t\right)=\overset{K}{\underset{k=1}{\sum }}a_{3}\left(\alpha_{k},\theta_{k}\right)s_{k}\left(t\right)+n_{3}\left(t\right)=A_{3}\left(\alpha ,\theta \right)s\left(t\right)+n_{3}\left(t\right).$$

where s(t) is the source signal vector. $n_{1}\left(t\right)$, $n_{2}\left(t\right)$ and $n_{3}\left(t\right)$ are noise vectors, all of which are assumed to be temporally and spatially independent and identically complex Gaussian distributed with zero mean and covariance $\sigma_{n}^{2}$. $A_{i}\left(\alpha ,\theta \right)=\left[a_{i}\left(\alpha_{1},\theta_{1}\right),\cdots ,a_{i}\left(\alpha_{K},\theta_{K}\right)\right],i=1,2,3$ is the manifold matrix of *i*-th subarray and $a_{i}\left(\alpha_{k},\theta_{k}\right)$ is the corresponding steering vector, i.e.,

(4) $$a_{1}\left(\alpha_{k},\theta_{k}\right)={\bar{a}}_{1}\left({\bar{\alpha }}_{k}\right)={\left[1,e^{j2\pi y_{1}{\bar{\alpha }}_{k}},\cdots ,e^{j2\pi y_{m}{\bar{\alpha }}_{k}},\cdots ,e^{j2\pi y_{\left(N_{1}-1\right)}{\bar{\alpha }}_{k}}\right]}^{T},$$

(5) $$a_{2}\left(\alpha_{k},\theta_{k}\right)={\bar{a}}_{2}\left({\bar{\alpha }}_{k}\right)e^{j2\pi {\bar{\theta }}_{k}}={\left[1,e^{j2\pi y_{1}{\bar{\alpha }}_{k}},\cdots ,e^{j2\pi y_{n}{\bar{\alpha }}_{k}},\cdots ,e^{j2\pi y_{\left(N_{2}-1\right)}N_{1}{\bar{\alpha }}_{k}}\right]}^{T}e^{j2\pi {\bar{\theta }}_{k}},$$

(6) $$a_{3}\left(\alpha_{k},\theta_{k}\right)={\bar{a}}_{3}\left({\bar{\alpha }}_{k}\right)e^{-j2\pi {\bar{\theta }}_{k}}={\left[1,e^{j2\pi y_{1}{\bar{\alpha }}_{k}},\cdots ,e^{j2\pi y_{n}{\bar{\alpha }}_{k}},\cdots ,e^{j2\pi y_{\left(N_{2}-1\right)}N_{1}{\bar{\alpha }}_{k}}\right]}^{T}e^{-j2\pi {\bar{\theta }}_{k}}.$$

where ${\bar{\alpha }}_{k}=\frac{d}{\lambda }\sin\left(\theta_{k}\right)\sin\left(\alpha_{k}\right)$ and ${\bar{\theta }}_{k}=\frac{d}{\lambda }\sin\left(\theta_{k}\right)\cos\left(\alpha_{k}\right)$, the same as the definitions as [25], and denote the normalized azimuth angle and normalized elevation angle of the *k*-th signal, respectively. Let $B=\mathrm{diag}\left[e^{j2\pi {\bar{\theta }}_{1}},\cdots ,e^{j2\pi {\bar{\theta }}_{K}}\right]$. Due to the identical *y*-axis coordinates of subarray2 and subarray3, ${\bar{a}}_{2}\left({\bar{\alpha }}_{k}\right)={\bar{a}}_{3}\left({\bar{\alpha }}_{k}\right)$. Thus, the manifold matrices of the three subarrays can be respectively expressed as

(7) $$A_{1}\left(\alpha ,\theta \right)={\bar{A}}_{1}\left(\bar{\alpha }\right)=\left[{\bar{a}}_{1}\left({\bar{\alpha }}_{1}\right),\cdots ,{\bar{a}}_{1}\left({\bar{\alpha }}_{K}\right)\right],$$

(8) $$A_{2}\left(\alpha ,\theta \right)={\bar{A}}_{2}\left(\bar{\alpha }\right)B=\left[{\bar{a}}_{2}\left({\bar{\alpha }}_{1}\right),\cdots ,{\bar{a}}_{2}\left({\bar{\alpha }}_{K}\right)\right]B,$$

(9) $$A_{3}\left(\alpha ,\theta \right)={\bar{A}}_{3}\left(\bar{\alpha }\right)B^{*}=\left[{\bar{a}}_{2}\left({\bar{\alpha }}_{1}\right),\cdots ,{\bar{a}}_{2}\left({\bar{\alpha }}_{K}\right)\right]B^{*}.$$

## 3. The Proposed Three-Parallel Nested Subarrays and 2-D DOA Estimation Method

In this section, we first propose two kinds of novel sparse arrays geometries to obtain the hole-free virtual arrays. They can be used to detect more sources with the same number of sensors as parallel co-prime array and its modifications. Then, a sparse representation framework is proposed to estimate the normalized azimuth angles and total least squares technique is proposed to estimate the corresponding normalized elevation angles accurately and automatically.

### 3.1. The Configurations of TPNAs

As defined in [8], a nested array is comprised of two uniform linear subarrays, and one subarray contains $N_{1}$ sensors with an inter-element spacing d, while the other subarray contains $N_{2}$ sensors with an inter-element spacing $\left(N_{1}+1\right)d$. By vectorizing the auto-correlation matrix of received data in a nested array, a hole-free difference coarray is obtained naturally, and the DOFs in the difference coarray are significantly increased. Inspired by the nested array, we propose two kinds of TPNAs to detect more sources than the number of sensors in the 2-D DOA estimation problem.

To obtain the hole-free coarray, we consider the configuration of the first TPNAs, named as TPNAs-I, which contains $N_{1}+2N_{2}$ sensors. As shown in Figure 1, it is assumed that subarray1 contains $N_{1}$ sensors with predefined inter-element spacing, whereas subarray2 and subarray3 respectively contain $N_{2}$ sensors with inter-element spacing $N_{1}d$. Specifically, the sensors’ x-axis coordinates in subarray1 are equal to zero, while those of subarray2 are equal to d and those of subarray3 are equal to −d. The sensor positions of subarray1 along the y-axis can be written as

(10) $$Y_{1}=Y_{1,1}\cup Y_{1,2}.$$

where $Y_{1,1}$ and $Y_{1,2}$ denote the first part and second part of subarray1, respectively, and can be expressed as

(11) $$Y_{1,1}=\{\begin{array}{c} \left\{y_{m}d|y_{m}=0,2,\cdots ,N_{1}-2\right\},\;\;N_{1}\;\mathrm{is}\;\mathrm{even}\\ \left\{y_{m}d|y_{m}=0,2,\cdots ,N_{1}-1\right\},\;\;N_{1}\;\mathrm{is}\;\mathrm{odd} \end{array},$$

(12) $$Y_{1,2}=\{\begin{array}{c} \left\{\left(-y_{n}+N_{1}\left(N_{2}-1\right)\right)d|y_{n}=1,3,\cdots ,N_{1}-1\right\},\;\;N_{1}\;\mathrm{is}\;\mathrm{even}\\ \left\{\left(-y_{n}+N_{1}\left(N_{2}-1\right)\right)d|y_{n}=1,3,\cdots ,N_{1}-2\right\},\;\;N_{1}\;\mathrm{is}\;\mathrm{odd} \end{array}.$$

Meanwhile, the sensor positions of subarray2 and subarray3 along the y-axis can be respectively written as

(13) $$Y_{2}=\left\{y_{2}N_{1}d|y_{2}=0,1,\cdots ,N_{2}-1\right\},$$

(14) $$Y_{3}=\left\{y_{3}N_{1}d|y_{3}=0,1,\cdots ,N_{2}-1\right\}.$$

As a result, the cross difference set of $Y_{1}$ and $Y_{2}$ and that of $Y_{3}$ and $Y_{1}$ can be respectively expressed as

(15) $$L_{12}=\left\{p_{i}-p_{j}|p_{i}\in Y_{1},p_{j}\in Y_{2}\right\},$$

(16) $$L_{31}=\left\{p_{i}-p_{j}|p_{i}\in Y_{3},p_{j}\in Y_{1}\right\}.$$

Thus, we have the union of sets $L_{12}$ and $L_{31}$ as

(17) $$L=L_{12}\cup L_{31},$$

where $L_{12}$ and $L_{31}$ respectively denote the cross difference coarrays with more elements than $Y_{1}$, $Y_{2}$ and $Y_{3}$. Their union extends the degrees of freedom significantly and can be denoted as the hole-free 1-D augmented virtual array, which can be used to detect more sources than the number of physical sensors with an appropriate 2-D DOA estimation method. By removing the repeated elements of L, we obtain the continuous augmented virtual array as

(18) $$L_{0}=\left\{-\left(N_{2}-1\right)N_{1}d,\cdots ,\left(N_{2}-1\right)N_{1}d\right\}.$$

Accordingly, we obtain the following theorem, which gives a summarization of TPNAs-I according to the aforementioned statements.

**Theorem** **1.** *Given a 2-D physical array with* $N_{1}+2N_{2}$ *sensors as illustrated in Figure 1, in which the sensor positions along the y-axis consist of* $Y_{1}$ *,* $Y_{2}$ *and* $Y_{3}$ *, by cross difference operation in (15) and (16), the hole-free 1-D augmented virtual array defined as (18) is then obtained, which can obtain* $2N_{1}N_{2}-2N_{1}+1$ *continuous DOFs.*

**Proof.**  See Appendix A. □

Meanwhile, in order to maximize $2N_{1}N_{2}-2N_{1}+1$ with the given number of sensors N, one should solve the following optimization problem:

(19) $$\begin{array}{c} \underset{N_{1},N_{2}\in {\mathbb{Z}}^{+}}{\max}2N_{1}N_{2}-2N_{1}+1\\ \mathrm{subject}\;\mathrm{to}:N=N_{1}+2N_{2} \end{array}.$$

It is easy to obtain the optimal parameters that $N_{2}=\left[\frac{N+2}{4}\right]$, $N_{1}=N-2N_{2}$. Specifically, the solutions of $N_{1}$ and $N_{2}$, as well as the achievable DOFs for TPNAs-I, can be verified as seen in Table 1. One needs to note that $N_{1}$ and $N_{2}$ have two different solutions to achieve the same continuous DOFs in the case of N=4k.

**Figure 2:**
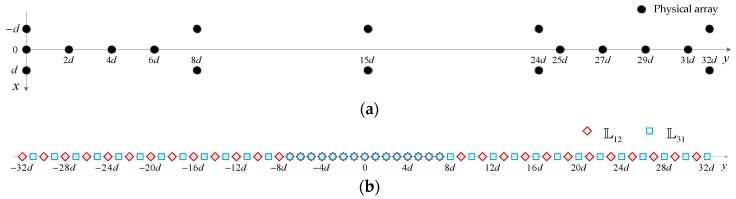
An example of TPNAs-I, where $N_{1}=8$, $N_{2}=5$. (**a**) physical array; (**b**) the corresponding difference coarray consist of $L_{12}$ and $L_{31}$.

To have some direct feeling for TPNAs-I, let us consider an example with 18 sensors in Figure 2. Accordingly, we have $N_{1}=8$ and $N_{2}=5$, the number of continuous DOFs of TPNAs-I as illustrated in Figure 2b is the largest and is equal to 65.

Please note that in Figure 2b, there are repeated elements from −7d to 7d between $L_{12}$ and $L_{31}$. This means the configuration of TPNAs-I still has certain improvable properties. In order to avoid the occurrence of repeated elements as much as possible between $L_{12}$ and $L_{31}$, we propose a second TPNAs, named as TPNAs-II, in which only sensor positions of subarray1 are rearranged along the y-axis; then, the sensors’ positions along the y-axis in subarray1 can be rewritten as

(20) $${\tilde{Y}}_{1}={\tilde{Y}}_{1,1}\cup {\tilde{Y}}_{1,2}.$$

where ${\tilde{Y}}_{1,1}$ and ${\tilde{Y}}_{1,2}$ denote the first part and second part of subarray1, respectively, and can be rewritten as

(21) $${\tilde{Y}}_{1,1}=\{\begin{array}{c} \left\{-y_{m}d|y_{m}=0,2,\cdots ,N_{1}-2\right\},\;\;N_{1}\;\mathrm{is}\;\mathrm{even}\\ \left\{-y_{m}d|y_{m}=0,2,\cdots ,N_{1}-1\right\},\;\;N_{1}\;\mathrm{is}\;\mathrm{odd} \end{array},$$

(22) $${\tilde{Y}}_{1,2}=\{\begin{array}{c} \left\{\left(y_{n}+N_{1}\left(N_{2}-1\right)\right)d|y_{n}=1,3,\cdots ,N_{1}-1\right\},\;\;N_{1}\;\mathrm{is}\;\mathrm{even}\\ \left\{\left(y_{n}+N_{1}\left(N_{2}-1\right)\right)d|y_{n}=1,3,\cdots ,N_{1}-2\right\},\;\;N_{1}\;\mathrm{is}\;\mathrm{odd} \end{array}.$$

Substituting (20) into (15) and (16), the corresponding union set of cross difference coarrays $L_{12}$ and $L_{31}$, after removing the repeated elements, can be expressed as

(23) $${\tilde{L}}_{0}=\left\{-\left(N_{1}N_{2}-1\right)d,\cdots ,\left(N_{1}N_{2}-1\right)d\right\}.$$

Since the rearrangement of subarray1, there exists only one repeated element in the coarray. Thus, we have the following theorem.

**Theorem** **2.** *Given a 2-D physical array with* $N_{1}+2N_{2}$ *sensors as illustrated in Figure 1, where the sensor positions along the y-axis consist of* ${\tilde{Y}}_{1}$ *,* $Y_{2}$ *and* $Y_{3}$ *, by cross difference operation in (15) and (16), the hole-free 1-D augmented virtual array defined as (18) is then obtained, which can obtain* $2N_{1}N_{2}-1$ *continuous DOFs.*

**Proof.**  The proof of Theorem 2 has a similar procedure to the proof of Theorem 1. So it is omitted. □

In this way, the corresponding optimization problem of DOFs in (19) can be expressed as

(24) $$\begin{array}{c} \underset{N_{1},N_{2}\in {\mathbb{Z}}^{+}}{\max}2N_{1}N_{2}-1\\ \mathrm{subject}\;\mathrm{to}:N=N_{1}+2N_{2} \end{array}.$$

Thus, we obtain the optimal parameters that $N_{2}=\left[\frac{N}{4}\right]$, $N_{1}=N-2N_{2}$. The relationship between N, $N_{1}$, $N_{2}$ and the DOFs of TPNAs-II are listed in Table 2. Similarly, $N_{1}$ and $N_{2}$ also have two different solutions to achieve the same continuous DOFs in the case of N=4k+2.

**Figure 3:**
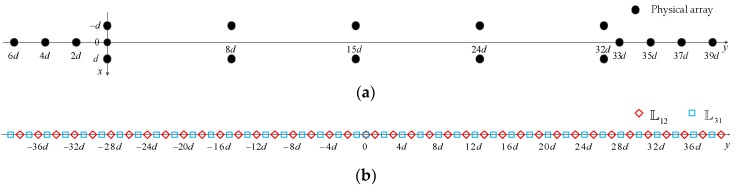
An example of TPNAs-II, where $N_{1}=8$, $N_{2}=5$. (**a**) physical array; (**b**) the corresponding difference coarray consisting of $L_{12}$ and $L_{31}$.

Meanwhile, an example of TPNAs-II is offered in Figure 3, where $N_{1}=8$ and $N_{2}=5$. With the same number of physical sensors as TPNAs-I in Figure 2, however, the continuous DOFs of TPNAs-II in this example increases to 79.

### 3.2. 2-D DOA Estimation Using Cross-Correlation Matrix via Sparse Representation

In this section, we assume that TPNAs-I is used to obtain the array measurement data. According to array measurement vectors (1)–(3) and the corresponding manifold matrices (7)–(9), the cross-correlation matrices between $x_{1}\left(t\right)$ and $x_{2}\left(t\right)$, and between $x_{3}\left(t\right)$ and $x_{1}\left(t\right)$ can be respectively written as

(25) $$R_{12}=E\left[x_{1}\left(t\right)x_{2}^{H}\left(t\right)\right]={\bar{A}}_{1}\left(\bar{\alpha }\right)R_{ss}B^{H}{\bar{A}}_{2}^{H}\left(\bar{\alpha }\right)+E_{12},$$

(26) $$R_{31}=E\left[x_{3}\left(t\right)x_{1}^{H}\left(t\right)\right]={\bar{A}}_{2}\left(\bar{\alpha }\right)B^{*}R_{ss}{\bar{A}}_{1}^{H}\left(\bar{\alpha }\right)+E_{31}.$$

where $R_{ss}=E\left[s\left(t\right)s^{H}\left(t\right)\right]=\mathrm{diag}\left[\sigma_{1}^{2},\sigma_{2}^{2},\cdots ,\sigma_{K}^{2}\right]$ is the K×K covariance matrix of the signals and $\sigma_{k}^{2}$ represents the power of *k*-th signal. According to the definition of B, we know that $B^{H}=B^{*}$. $E_{12}=0$, $E_{31}=0$ respectively represents the residual terms of $R_{12}$ and $R_{31}$. Please note that the theoretical cross-correlation matrices defined in (25) and (26) are unavailable in practical situations. They are usually approximated from the finite snapshot array measurement vectors as

(27) $${\hat{R}}_{12}=\frac{1}{T}\overset{T}{\underset{t=1}{\sum }}x_{1}\left(t\right)x_{2}^{H}\left(t\right)={\bar{A}}_{1}\left(\bar{\alpha }\right){\hat{R}}_{ss}B^{H}{\bar{A}}_{2}^{H}\left(\bar{\alpha }\right)+{\hat{E}}_{12},$$

(28) $${\hat{R}}_{31}=\frac{1}{T}\overset{T}{\underset{t=1}{\sum }}x_{3}\left(t\right)x_{1}^{H}\left(t\right)={\bar{A}}_{2}\left(\bar{\alpha }\right)B^{*}{\hat{R}}_{ss}{\bar{A}}_{1}^{H}\left(\bar{\alpha }\right)+{\hat{E}}_{31}.$$

where T is the number of snapshots, ${\hat{R}}_{ss}=\frac{1}{T}\sum_{t=1}^{T}s\left(t\right)s^{H}\left(t\right)$ is the estimated covariance matrix of signals. In addition, the approximated estimated values of ${\hat{E}}_{12}$ and ${\hat{E}}_{31}$ can be expressed as

(29) $${\hat{E}}_{12}=\frac{{\bar{A}}_{1}\left(\bar{\alpha }\right)}{T}\overset{T}{\underset{t=1}{\sum }}s\left(t\right)n_{2}^{H}\left(t\right)+\left[\overset{T}{\underset{t=1}{\sum }}n_{1}\left(t\right)s^{H}\left(t\right)\right]\frac{B^{H}{\bar{A}}_{2}\left(\bar{\alpha }\right)}{T}+\frac{1}{T}\overset{T}{\underset{t=1}{\sum }}n_{1}\left(t\right)n_{2}^{H}\left(t\right),$$

(30) $${\hat{E}}_{31}=\frac{{\bar{A}}_{2}\left(\bar{\alpha }\right)B^{*}}{T}\overset{T}{\underset{t=1}{\sum }}s\left(t\right)n_{1}^{H}\left(t\right)+\left[\overset{T}{\underset{t=1}{\sum }}n_{3}\left(t\right)s^{H}\left(t\right)\right]\frac{{\bar{A}}_{1}\left(\bar{\alpha }\right)}{T}+\frac{1}{T}\overset{T}{\underset{t=1}{\sum }}n_{3}\left(t\right)n_{1}^{H}\left(t\right).$$

By vectorizing the cross-correlation matrix $R_{12}$ in (25), we can obtain a long vector that can be regarded as the received data from a virtual array with extended coarray aperture, i.e.,

(31) $$r_{12}=\mathrm{vec}\left(R_{12}\right)=C_{12}\left(\bar{\alpha }\right)p+z_{12}.$$

where $C_{12}\left(\bar{\alpha }\right)=\left[{\bar{a}}_{2}^{*}\left({\bar{\alpha }}_{1}\right)⊗{\bar{a}}_{1}\left({\bar{\alpha }}_{1}\right),{\bar{a}}_{2}^{*}\left({\bar{\alpha }}_{2}\right)⊗{\bar{a}}_{1}\left({\bar{\alpha }}_{2}\right),\cdots ,{\bar{a}}_{2}^{*}\left({\bar{\alpha }}_{K}\right)⊗{\bar{a}}_{1}\left({\bar{\alpha }}_{K}\right)\right]$ is the manifold matrix of this virtual array, $p={\left[\sigma_{1}^{2}e^{-j2\pi {\bar{\theta }}_{1}},\sigma_{2}^{2}e^{-j2\pi {\bar{\theta }}_{2}},\cdots ,\sigma_{K}^{2}e^{-j2\pi {\bar{\theta }}_{K}}\right]}^{T}$ is a single snapshot signal vector and $z_{12}=\mathrm{vec}\left(E_{12}\right)$. With the observation of (31), we know that a virtual array is generated naturally by the cross-correlation matrix vectorization operation, and its sensor positions are denoted as

(32) $$D_{12}=L_{12}=\left\{p_{i}-p_{j}|p_{i}\in Y_{1},p_{j}\in Y_{2}\right\}.$$

Similarly, the vectorized cross-correlation matrix of $R_{31}$ with the characteristic of extended coarray aperture can be expressed as

(33) $$r_{31}=\mathrm{vec}\left(R_{31}\right)=C_{31}\left(\bar{\alpha }\right)p+z_{31}.$$

where $C_{31}\left(\bar{\alpha }\right)=\left[{\bar{a}}_{1}^{*}\left({\bar{\alpha }}_{1}\right)⊗{\bar{a}}_{2}\left({\bar{\alpha }}_{1}\right),{\bar{a}}_{1}^{*}\left({\bar{\alpha }}_{2}\right)⊗{\bar{a}}_{2}\left({\bar{\alpha }}_{2}\right),\cdots ,{\bar{a}}_{1}^{*}\left({\bar{\alpha }}_{K}\right)⊗{\bar{a}}_{2}\left({\bar{\alpha }}_{K}\right)\right]$ and $z_{31}=\mathrm{vec}\left(E_{31}\right)$. The corresponding virtual sensor positions are denoted as

(34) $$D_{31}=L_{31}=\left\{p_{i}-p_{j}|p_{i}\in Y_{3},p_{j}\in Y_{1}\right\}.$$

According to (31) and (33), we define a new data vector as follows

(35) $$r=\left[\begin{array}{c} r_{12}\\ r_{31} \end{array}\right]=C\left(\bar{\alpha }\right)p+z.$$

where $C\left(\bar{\alpha }\right)={\left[C_{12}{\left(\bar{\alpha }\right)}^{T},C_{31}{\left(\bar{\alpha }\right)}^{T}\right]}^{T}$, $z={\left[z_{12}^{T},z_{31}^{T}\right]}^{T}$. As such, an augmented virtual array with enhanced DOFs is obtained, whose sensor locations are given by

(36) $$D=D_{12}\cup D_{31}=L_{12}\cup L_{31}.$$

Interestingly, Equation (32) is the cross difference set of $Y_{1}$ and $Y_{2}$, Equation (34) is the cross difference set of $Y_{3}$ and $Y_{1}$, while Equation (36) is the combination of (32) and (34). As a result, the DOFs of (36) can theoretically be increased by up to twice $D_{12}$ or $D_{31}$. By removing the repeated entries in D and sorting them in ascending order, then extracting the corresponding entries from vector r, we obtain the augmented virtual received data vector, i.e.,

(37) $$r_{0}=C_{0}\left(\bar{\alpha }\right)p+z_{0}.$$

and the augmented virtual array can be expressed as

(38) $$D_{0}=L_{0}=\left\{-\left(N_{2}-1\right)N_{1}d,\cdots ,\left(N_{2}-1\right)N_{1}d\right\}.$$

Please note that the augmented virtual received data vector $r_{0}$ is a single snapshot in the coarray domain, and the equivalent virtual source signal vector p behaves like fully coherent sources [8,32]; the DOAs thus cannot be effectively estimated by applying the subspace-based DOA estimation techniques directly in this case. However, DOA estimation methods via the sparse representation framework can handle the coherent sources problem naturally, which are efficient for the single snapshot condition. By discretizing the normalized azimuth angle range into finite sampling grids ${\bar{\alpha }}^{g}=\left\{{\bar{\alpha }}_{1}^{g},{\bar{\alpha }}_{2}^{g},\cdots ,{\bar{\alpha }}_{L}^{g}\right\}$, where L≫K, the augmented virtual received data vector can be sparsely represented over the entire discretized angular girds as

(39) $$r_{0}=C_{0}\left({\bar{\alpha }}^{g}\right)p^{g}+z_{0}.$$

where $C_{0}\left({\bar{\alpha }}^{g}\right)=\left[c_{0}\left({\bar{\alpha }}_{1}^{g}\right),c_{0}\left({\bar{\alpha }}_{2}^{g}\right),\cdots ,c_{0}\left({\bar{\alpha }}_{L}^{g}\right)\right]$ is defined as the collection of steering vectors in the augmented virtual array domain over the finite sampling grids, the positions of non-zero entries in $p^{g}$ represent the DOA estimations and that values denote the corresponding coefficients. Then, one can model the sparse representation framework-based DOA estimation as

(40) $$\begin{array}{c} \underset{{\hat{p}}^{g}\in {\mathbb{C}}^{L}}{\min}\;\;\;\;\|{\hat{p}}^{g}{}_{1}\|\\ \mathrm{subject}\;\mathrm{to}:||r_{0}-C_{0}\left({\bar{\alpha }}^{g}\right){\hat{p}}^{g}||{}_{2}<\eta \end{array}.$$

where η is a regularization parameter which can be used to trade off the sparsity of ${\hat{p}}^{g}$ and the representation error of $r_{0}$. Then, the Equation (40) can be transformed into an unconstrained optimization problem [33] as

(41) $$\underset{{\hat{p}}^{g}\in {\mathbb{C}}^{L}}{\min\;}\;\;\left[\frac{1}{2}||r_{0}-C_{0}\left({\bar{\alpha }}^{g}\right){\hat{p}}^{g}||{}_{2}+\eta \;||{\hat{p}}^{g}||{}_{1}\right].$$

Please note that the value of η needs to be selected properly so as to approximately match the statistics of the noise term [26,34]. In practical applications, when finite snapshot array measurement vectors are used to estimate the sample cross-correlation matrices, the residual terms in (25) and (26) can also be estimated from (29) and (30). In low signal-to-noise ratio (SNR) situations, ${\hat{E}}_{12}$ and ${\hat{E}}_{31}$ can be represented approximately by the last terms of themselves, respectively. In addition, it shows that the entries of ${\hat{E}}_{12}$ and ${\hat{E}}_{31}$ satisfy an asymptotic complex Gaussian distribution, i.e., $\hat{e}\sim \mathrm{AsCN}\left(0,\frac{\sigma_{n}^{4}}{4}\right)$, where $\hat{e}$ is an arbitrary entry of ${\hat{E}}_{12}$ and ${\hat{E}}_{31}$. Accordingly, the distribution of entries of $z_{0}$ can be expressed approximately as

(42) $$\frac{T}{\sigma_{n}^{4}}\overset{\mathrm{DOFs}}{\underset{i=1}{\sum }}z_{i}^{2}\sim As\chi^{2}\left(\mathrm{DOFs}\right).$$

where $As\chi^{2}\left(\mathrm{DOFs}\right)$ is the asymptotic Chi-square distribution. Then, the regularization parameter should be chosen as

(43) $$\sqrt{\overset{\mathrm{DOFs}}{\underset{i=1}{\sum }}z_{i}^{2}}\leq \eta .$$

holds with a high probability.

Once we have obtained the estimations of ${\hat{\bar{\alpha }}}_{k},k=1,2,\cdots ,K$ according to the estimated vector ${\hat{p}}^{g}$ from (41), the manifold matrix of augmented virtual array can be reconstructed as ${\hat{C}}_{0}\left(\hat{\bar{\alpha }}\right)=\left[{\hat{c}}_{0}\left({\hat{\bar{\alpha }}}_{1}\right),{\hat{c}}_{0}\left({\hat{\bar{\alpha }}}_{2}\right),\cdots ,{\hat{c}}_{0}\left({\hat{\bar{\alpha }}}_{K}\right)\right]$. Due to the finite snapshot sampling, there are errors existing in the augmented virtual received data vector $r_{0}$ and its corresponding estimated manifold matrix ${\hat{C}}_{0}\left(\hat{\bar{\alpha }}\right)$. The total least squares technique [28,35] which has been demonstrated efficient in the errors-in-variables linear regression model, can be used in Equation (37) to estimate the equivalent virtual source signal vector p. Accordingly, we can obtain the solution of the equivalent virtual source signal vector p as

(44) $$\hat{p}={\left({\hat{C}}_{0}^{H}{\hat{C}}_{0}-I_{K}\right)}^{-1}{\hat{C}}_{0}^{H}r_{0}.$$

Then, the estimations of ${\hat{\bar{\theta }}}_{k},k=1,2,\cdots ,K$ can be given by

(45) $${\hat{\bar{\theta }}}_{k}=-\frac{\arg\left({\hat{p}}_{k}\right)}{2\pi }.$$

where ${\hat{p}}_{k}$ is the *k*-th element of $\hat{p}$ and $\arg\left({\hat{p}}_{k}\right)$ represents the argument of ${\hat{p}}_{k}$. Obviously, the estimated parameter of ${\hat{\bar{\theta }}}_{k}$ is paired automatically with ${\hat{\bar{\alpha }}}_{k}$.

From the above analysis, the steps of 2-D DOA estimation using the proposed TPNAs-I via sparse representation are listed in Table 3. Please note that the steps in Table 3 are also applicable for the configuration of TPNAs-II.

## 4. Simulation Results

In this section, we provide several simulation experiments to illustrate the superiority of the proposed two kinds of TPNA and 2-D DOA estimation method. The performances of two kinds of TPNAs and 2-D DOA estimation method are compared with PCPA [27], S-CPPA [28] and TPCPA [29], as well as the respective 2-D DOA estimation method. What needs to be noticed is that in [27] and [29], the normalized elevation angles are obtained by a least squares technique, while the total least squares technique is used in [28] and our method, and an augmented matrix was constructed in [29] to obtain two snapshot virtual data vectors to enhance the estimation performance. The parameter of inter-element unit spacing was chosen to be equal to $\frac{\lambda }{2}$ for all array configurations.

### 4.1. DOF Comparison

**Figure 4:**
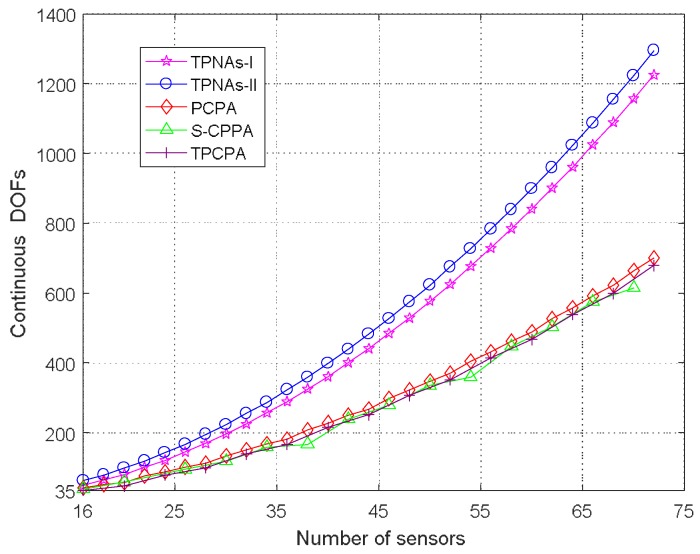
Continuous DOFs comparison for five kinds of physical array configurations.

We evaluate the continuous DOFs of the two proposed kinds of TPNAs in the first simulation and compare that with PCPA, S-CPPA and TPCPA. It needs to be noted that the proposed two kinds of TPNAs can offer $O\left(N^{2}\right)$ continuous DOFs with O(N) sensors, while PCPA, S-CPPA and TPCPA can offer O(MN) continuous DOFs with O(M+N) sensors, M and N are coprime integers. Figure 4 depicts the respective continuous DOFs with the number of sensors for five kinds of physical array configuration varying from 16 to 72. It is obvious that the continuous DOFs of the proposed two kinds of TPNAs achieve significant enhancement compared to the other three kinds of sparse arrays. Furthermore, due to the rearrangement of subarray1 sensor positions in TPNAs-II, the number of continuous DOFs of TPNAs-II is largest.

### 4.2. 2-D DOA Estimation Results

**Figure 5:**
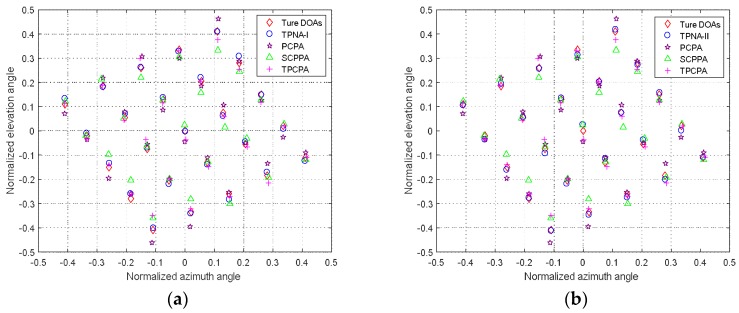
2-D DOA estimation results comparison, where T=500 and SNR=0 dB. (**a**) 2-D DOA estimation results of the proposed TPNAs-I and that of other three methods; (**b**) 2-D DOA estimation results of the proposed TPNAs-II and that of other three methods.

In the second experiment, we compared the 2-D DOA estimation results of five kinds of different array configurations and the respective 2-D DOA estimation method. The number of sensors of the five different array configurations were set as 18, i.e., $N_{1}=8$, $N_{2}=5$ for TPNAs-I and TPNAs-II, according to Section 3.1, M=5, N=8 for PCPA, M=3, N=4 for SCPPA and M=4, N=5 for TPCPA. Thus, we know that their continuous DOFs are respectively equal to 65, 79, 52, 47 and 47. This shows that, under the situation of the same number of sensors, the two proposed kinds of TPNAs and 2-D DOA estimation method can detect more sources than PCPA, SCPPA and TPCPA, as well as the corresponding 2-D DOA estimation method. Assuming 25 far-field narrowband uncorrelated sources illuminate at five different configurations, where the normalized angles of which are selected according to the reference [25]. The number of snapshots is set as T=500 and the SNR is set as 0 dB. The CVX toolbox [36] is used to solve the complex unconstrained minimization problem in (41), the regularization parameter η and the search step size are respectively set as 0.65 and 0.001. As shown in Figure 5a,b, the 2-D DOA estimation results of the five kinds of array configurations and their respective 2-D DOA estimation method are obtained.

Obviously, although all of the 2-D DOAs can be estimated successfully for all of the array configurations and respective 2-D DOA estimation method, the proposed two kinds of TPNAs and 2-D DOA estimation method show better detection performance. This validates the effectiveness of the proposed physical arrays configurations and 2-D DOA estimation method.

### 4.3. RMSE Performance

**Figure 6:**
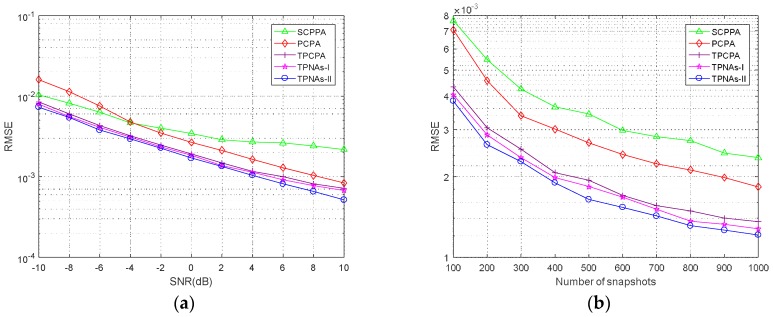
RMSE performance comparison with three signals. (**a**) As a function of SNR with T=500, (**b**) as a function of snapshots with SNR=0 dB.

In this subsection, we conduct 500 Monte Carlo trials to further study the empirical performance of the two proposed kinds of TPNAs and 2-D DOA estimation method. The RMSEs are computed to compare with the other three kinds of physical arrays configurations including PCPA, S-CPPA and TPCPA, and the respective 2-D DOA estimation method. The RMSE is defined as

$$\mathrm{RMSE}=\sqrt{\frac{1}{QK}\overset{Q}{\underset{q=1}{\sum }}\overset{K}{\underset{k=1}{\sum }}\left({\left({\hat{\bar{\alpha }}}_{k,q}-{\bar{\alpha }}_{k}\right)}^{2}+{\left({\hat{\bar{\theta }}}_{k,q}-{\bar{\theta }}_{k}\right)}^{2}\right)}.$$

where Q denotes the number of Monte Carlo trials and $\left({\hat{\bar{\alpha }}}_{k,q},{\hat{\bar{\theta }}}_{k,q}\right)$ is the estimation of the *k*-th source for the *q*-th trial. We consider the situation where three signals imping on the arrays. The normalized DOA of the first signal is randomly selected with a probability of uniform distribution from −0.2 to 0.2, then the other two normalized DOAs are set as $\left({\bar{\alpha }}_{2},{\bar{\theta }}_{2}\right)=\left({\bar{\alpha }}_{1}-0.2,{\bar{\theta }}_{1}-0.2\right)$ and $\left({\bar{\alpha }}_{3},{\bar{\theta }}_{3}\right)=\left({\bar{\alpha }}_{1}+0.2,{\bar{\theta }}_{1}+0.2\right)$, respectively. Meanwhile, the regularization parameter is set as 1.6 and the parameters of sensor number and search step size are set the same as those in Section 4.2. Figure 6a shows the RMSE performance as a function of SNR, where the number of snapshots is 500. In Figure 6b, we fix SNR=0 dB and compare the RMSE performance with respect to the number of snapshots. It is obvious from the RMSE curves in Figure 6 that the performance of the proposed two kinds of TPNAs and 2-D DOA estimation method outperform SCPPA and PCPA. The reason is that the apertures in the coarray domains of TPNAs-I and TPNAs-II are larger than those of SCPPA and PCPA. One needs to note that the RMSE results of the proposed two kinds of TPNAs and 2-D DOA estimation method in this article are comparable with TPCPA. This is due to the fact that the continuous DOFs of TPNAs-I and TPNAs-II possess the same order of magnitude. As for TPCPA, an augmented matrix was constructed in [29], which means that two snapshot virtual data vectors can be used to perform 2-D DOA estimation to enhance the system performance.

### 4.4. Resolution Ability

**Figure 7:**
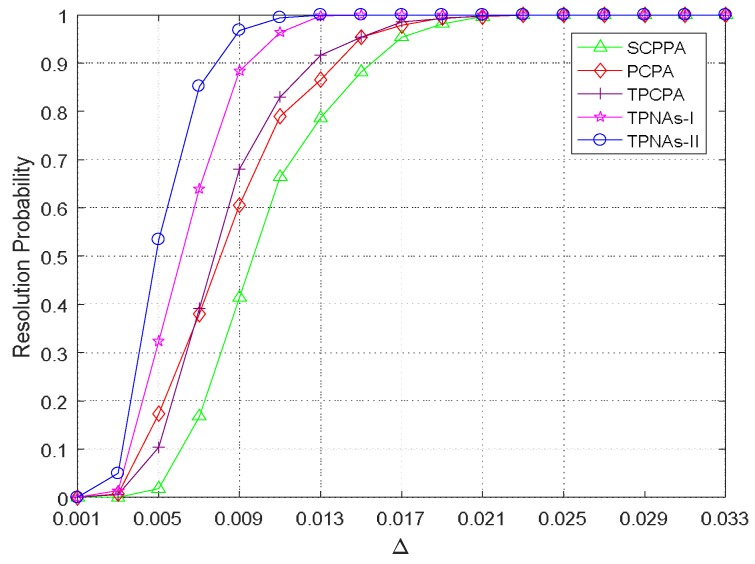
Resolution probability versus Δ, where T=500 and SNR=0 dB.

In this experiment, we evaluated the resolution ability of the proposed two kinds of physical arrays configurations and 2-D DOA estimation method and compared it with other three kinds of physical array configurations and respective 2-D DOA estimation methods. We assumed that there are two signals impinging on the arrays. The normalized 2-D DOA $\left({\bar{\alpha }}_{1},{\bar{\theta }}_{1}\right)$ was randomly selected with a probability of uniform distribution from −0.4 to 0.4, then the normalized 2-D DOA of the second signal is set as $\left({\bar{\alpha }}_{2},{\bar{\theta }}_{2}\right)=\left({\bar{\alpha }}_{1}+\Delta ,{\bar{\theta }}_{1}+\Delta \right)$. Thus, the normalized angular spacing can be defined as $\delta =\sqrt{2\Delta^{2}}$. When $\sqrt{{\left({\hat{\bar{\alpha }}}_{1}-{\bar{\alpha }}_{1}\right)}^{2}+{\left({\hat{\bar{\theta }}}_{1}-{\bar{\theta }}_{1}\right)}^{2}}<\frac{\delta }{2}$ and $\sqrt{{\left({\hat{\bar{\alpha }}}_{2}-{\bar{\alpha }}_{2}\right)}^{2}+{\left({\hat{\bar{\theta }}}_{2}-{\bar{\theta }}_{2}\right)}^{2}}<\frac{\delta }{2}$ in a trial, we consider two close signals to have been correctly resolved. Δ varies from 0.001 to 0.033 and the regularization parameter is set as 0.4. We select the number of snapshots and SNR respectively as 500 and 0 dB. The other parameters are the same as those in the previous experiment. Figure 7 shows the experiment results of resolution ability versus Δ with 500 Monte Carlo trials. It can be observed that the proposed two kinds of TPNAs and 2-D DOA estimation method show the significantly better performance than the other three sparse arrays and 2-D DOA estimation methods. The proposed TPNAs-II and 2-D DOA estimation method gets the highest resolution ability, followed by TPNAs-I, and the resolution ability of TPCPA is comparable with that of PCPA, while SCPPA shows the worst resolution ability. Obviously, the experiment results in Figure 7 are consistent with the continuous DOFs difference of the five kinds of sparse arrays configurations. In addition, compared to SCPPA, the performance improvement of resolution ability of TPCPA is due to the use of an augmented matrix.

## 5. Conclusions

In this paper, we have proposed two kinds of novel TPNAs and 2-D DOA estimation method for the 2-D DOA estimation problem. The proposed two kinds of TPNAs consist of three parallel subarrays that have $N_{1}$, $N_{2}$ and $N_{2}$ sensors with specific inter-element spacing, respectively. Due to the systematic design of TPNAs, they can achieve up to $O\left(N^{2}\right)$ continuous DOFs using only O(N) sensors. Accordingly, the two proposed kinds of TPNAs can detect more sources than there are sensors. In addition, sparse representation and the total least squares technique are used to estimate the 2-D DOAs based on the augmented virtual array. As a result, the obtained DOFs can be used fully for the 2-D DOA estimation problem. At last, simulation results showed the superiority and effectiveness of the two proposed kinds of sparse arrays configurations and 2-D DOA estimation method.

## Figures and Tables

**Table 1:** The relationship between N, $N_{1}$, $N_{2}$ and DOFs of TPNAs-I.

N (k Is an Integer)	$N_{1}$	$N_{2}$	Continuous DOFs
4k	2k or 2k−2	k or k+1	$\frac{N^{2}}{4}-N+1$
4k+1	2k−1	k+1	$\frac{N^{2}}{4}-N+\frac{7}{4}$
4k+2	2k	k+1	$\frac{N^{2}}{4}-N+2$
4k+3	2k+1	k+1	$\frac{N^{2}}{4}-N+\frac{7}{4}$

**Table 2:** The relationship between N, $N_{1}$, $N_{2}$ and DOFs of TPNAs-II.

N (k Is an Integer)	$N_{1}$	$N_{2}$	Continuous DOFs
4k	2k	k	$\frac{N^{2}}{4}-1$
4k+1	2k+1	k	$\frac{N^{2}}{4}-\frac{5}{4}$
4k+2	2k+2 or 2k	k or k+1	$\frac{N^{2}}{4}-2$
4k+3	2k+1	k+1	$\frac{N^{2}}{4}-\frac{5}{4}$

**Table 3:** Steps for 2-D DOA estimation method using TPNAs-I.

**Input**	The array measurement vectors $x_{1}\left(t\right)$, $x_{2}\left(t\right)$ and $x_{3}\left(t\right)$ with T snapshots.
**Output**	The estimations of normalized 2-D DOAs.
**Step1**	Calculate the cross-correlation matrices by (27) and (28).
**Step2**	Construct two received data vectors of virtual array by (31) and (33).
**Step3**	Obtain the augmented virtual received data vector by (35) and (37).
**Step4**	Select the regularization parameter according to (43).
**Step5**	Discretizing the normalized azimuth angle range and perform Equation (41) to obtain the estimations of ${\hat{\bar{\alpha }}}_{k},k=1,2,\cdots ,K$.
**Step6**	Calculate the estimations of ${\hat{\bar{\theta }}}_{k},k=1,2,\cdots ,K$ by (44) and (45).

## Appendix A. Proof of Theorem 1

Consider a case where $N_{1}$ is even, by substituting (10) and (13) into (15), we have

(A1) $$\begin{array}{ll} L_{12} & =\left\{\left(y_{m}-y_{2}N_{1}\right)d\right\}\cup \left\{\left(\left(-y_{n}+N_{1}\left(N_{2}-1\right)\right)-y_{2}N_{1}\right)d\right\}\\ & =\left\{\left(y_{m}-y_{2}N_{1}\right)d\right\}\cup \left\{\left(\left(N_{2}-1-y_{2}\right)N_{1}-y_{n}\right)d\right\}\\ & =\underset{U_{1}}{\underbrace{\left\{\left(y_{m}-y_{2}N_{1}\right)d\right\}}}\cup \underset{V_{1}}{\underbrace{\left\{\left(v_{1}N_{1}-y_{n}\right)d\right\}}} \end{array}$$

Similarly, $L_{31}$ can be expressed as

(A2) $$\begin{array}{ll} L_{31} & =\left\{\left(y_{3}N_{1}-y_{m}\right)d\right\}\cup \left\{\left(y_{3}N_{1}-\left(-y_{n}+N_{1}\left(N_{2}-1\right)\right)\right)d\right\}\\ & =\left\{\left(y_{3}N_{1}-y_{m}\right)d\right\}\cup \left\{\left(y_{n}-\left(N_{2}-1-y_{3}\right)N_{1}\right)d\right\}\\ & =\underset{U_{2}}{\underbrace{\left\{\left(y_{3}N_{1}-y_{m}\right)d\right\}}}\cup \underset{V_{2}}{\underbrace{\left\{\left(y_{n}-v_{2}N_{1}\right)d\right\}}} \end{array}$$

where $y_{m}=0,2,\cdots ,N_{1}-2$, $y_{n}=1,3,\cdots ,N_{1}-1$ and $y_{2}=y_{3}=0,1,\cdots ,N_{2}-1$. Evidently, we have $v_{1}=v_{2}=y_{2}=y_{3}\in \left\{0,1,\cdots ,N_{2}-1\right\}$. The union of $L_{12}$ and $L_{31}$ in (17) can be expressed as

(A3) $$\begin{array}{ll} L & =L_{12}\cup L_{31}\\ & =\left(U_{1}\cup V_{1}\right)\cup \left(U_{2}\cup V_{2}\right)\\ & =\underset{U}{\underbrace{\left\{\left(u-vN_{1}\right)d\right\}}}\cup \underset{V}{\underbrace{\left\{\left(vN_{1}-u\right)d\right\}}} \end{array}.$$

where $u=0,1,\cdots ,N_{1}-1$, $v=0,1,\cdots ,N_{2}-1$. Then, by unfolding the sets U and V, one can obtain

(A4) $$U=\left\{-\left(N_{2}-1\right)N_{1}d,\cdots ,0,\cdots ,\left(N_{1}-1\right)d\right\},$$

(A5) $$V=\left\{-\left(N_{1}-1\right)d,\cdots ,0,\cdots ,\left(N_{2}-1\right)N_{1}d\right\}.$$

Obviously, U and V can be treated as uniform linear arrays with the inter-element spacing d. At the same time, the former’s sensor positions vary from $-\left(N_{2}-1\right)N_{1}d$ to $\left(N_{1}-1\right)d$, while the latter’s sensors positions vary from $-\left(N_{1}-1\right)d$ to $\left(N_{2}-1\right)N_{1}d$. Therefore, there exist repeated elements from $-\left(N_{1}-1\right)d$ to $\left(N_{1}-1\right)d$ between U and V. By removing the repeated elements between U and V, we get $L_{0}$. Consequently, we can draw the conclusion that the continuous DOFs of TPNAs-I are equal to $2N_{1}N_{2}-2N_{1}+1$.

In the case where $N_{1}$ is odd, we can draw an identical conclusion with a similar proof procedure to that above, which is omitted to avoid redundancy.
